# Identification and functional analysis of hub genes in knee osteoarthritis via bioinformatics and experimental validation

**DOI:** 10.3389/fbinf.2025.1671693

**Published:** 2025-12-17

**Authors:** Shanyong Jiang, Jingjing Cao, Jianshu Lu, Jianxiao Liang, Lianxin Li, Yanqiang Song, Jincheng Gao, Baoen Jiang

**Affiliations:** 1 Department of Orthopaedics, Dongying People’s Hospital, Dongying, Shandong, China; 2 Department of Radiology, Dongying People’s Hospital, Dongying, Shandong, China; 3 Department of Traumatic Orthopedics, Shandong Provincial Hospital Affiliated to Shandong First Medical University, Jinan, Shandong, China; 4 Department of Traditional Chinese Medicine, Dongying People’s Hospital, Dongying, Shandong, China

**Keywords:** knee osteoarthritis, bioinformatics, differentially expressed genes, immuneinfiltration, GSVA

## Abstract

**Objective:**

Knee osteoarthritis (KOA) is a prevalent chronic degenerative joint disease that causes chronic pain and mobility restrictions in the elderly, significantly impacting quality of life. Current treatments focus on symptom relief, lacking effective interventions targeting the underlying mechanisms. Understanding KOA’s molecular mechanisms and identifying key pathogenic genes are essential for developing targeted therapies.

**Methods:**

Gene expression data from KOA patients and healthy controls were obtained from the Gene Expression Omnibus (GEO) database, and differentially expressed genes (DEGs) were identified. Gene Ontology (GO) and Kyoto Encyclopedia of Genes and Genomes (KEGG) enrichment analyses were performed to reveal the associated biological processes and signaling pathways. Protein-protein interaction (PPI) network analysis and Gene Ontology-based semantic similarity calculations were used to identify hub genes. Gene Set Variation Analysis (GSVA) assessed enrichment in KOA-related pathways. Immune infiltration analysis (CIBERSORT) assessed the immune cell distribution in KOA tissues. Finally, hub gene expression changes were validated using the IL-1β-treated CHON-001 cell model and real-time quantitative PCR (RT-qPCR).

**Results:**

A total of 3,290 upregulated and 2,536 downregulated DEGs were identified. GO and KEGG enrichment analyses revealed these genes were primarily involved in extracellular matrix remodeling, transmembrane transport, and inflammation-related pathways. Key hub genes, including HSPA5, FOXO1, and YWHAE, were identified. GSVA showed that these genes were significantly enriched in multiple KOA-associated signaling pathways. Immune infiltration analysis revealed significant differences in the levels of six immune cell types in KOA tissues, which were associated with the hub genes expression. In CHON-001 cell, the expression levels of GRB2, IKBKG, and HSPA12A were upregulated, whereas YWHAE, HSPB1, and DCAF8 were downregulated, consistent with the tissue samples.

**Conclusion:**

This study identified key pathogenic genes and their regulatory pathways in KOA, highlighting their potential role in disease progression via inflammation and immune modulation. These findings provide insights for developing targeted therapeutic strategies for KOA.

## Introduction

Knee osteoarthritis (KOA) is a chronic, progressive joint disease characterized by degenerative changes in articular cartilage, synovial inflammation, and subchondral bone remodeling (Ungsudechachai et al., 2021; Hu et al., 2021). With the global aging population, its prevalence continues to rise, currently accounting for approximately 85% of osteoarthritis (OA) cases worldwide. The incidence of symptomatic KOA is estimated to be 8.1% (Li et al., 2020). Typical clinical manifestations of KOA include joint pain, stiffness, limited mobility, and joint deformity (Surgeons et al., 2021). In severe cases, patients may experience weight-bearing difficulties or even disability, affecting daily living activities and potentially resulting in adverse effects on mental health (Turner et al., 2020).

KOA has a complex etiology, significant heterogeneity, and a slow disease progression with considerable inter-individual variability (Luo et al., 2024). Prolonged abnormal mechanical loading, such as obesity, knee malalignment, history of trauma, and occupational overuse, is one of the primary risk factors for KOA, leading to microdamage and structural deterioration of cartilage (Wang et al., 2024; Messier et al., 2014; Otsuki et al., 2016). In addition, inflammatory responses, dysregulated immune regulation, genetic susceptibility, and metabolic disorders act synergistically in the onset and progression of KOA (Huang et al., 2024). Due to the lack of vascular and neural supply, articular cartilage has extremely limited regenerative capacity; once damaged, its ability to repair is minimal, resulting in joint dysfunction and further disease progression (He et al., 2019; Abramoff and Caldera, 2020).

Currently, total knee arthroplasty (TKA) remains the primary treatment for pain relief and functional improvement in patients with end-stage KOA (Xu et al., 2023). For patients in the early to mid-stages of KOA, treatment primarily consists of non-surgical conservative approaches, including nonsteroidal anti-inflammatory drugs (NSAIDs), physical therapy, exercise rehabilitation, and intra-articular injections, aiming to slow disease progression (Thorlund et al., 2022). In recent years, with advances in cell biology, molecular biology, and omics technologies, research on the molecular mechanisms of KOA has deepened. Increasing attention has been given to the potential applications of biomarkers in early diagnosis, treatment monitoring, and targeted therapy (Boffa et al., 2021). A study utilizing a chondrocyte injury model induced by interleukin-1β (IL-1β) demonstrated that exosomes derived from human urine-derived stem cells overexpressing miR-140-5p (hUSC-140-Exos) could promote chondrocyte proliferation and migration, inhibit apoptosis, and enhance the synthesis of extracellular matrix (ECM) components. *In vivo*, administration of hUSC-140-Exos in a rat KOA model resulted in improved cartilage regeneration and subchondral bone remodeling, exhibiting significant chondroprotective effects (Liu et al., 2022). Regarding therapeutic strategies, studies have also shown that traditional Chinese medicine formulations may upregulate the long non-coding RNA UFC1, thereby inhibiting miR-34a activity and reducing matrix metalloproteinase-13 (MMP-13) expression, ultimately delaying cartilage degeneration (Zhao et al., 2025). Therefore, investigating the molecular mechanisms of KOA and identifying key pathogenic genes are crucial for the development of novel targeted therapeutic strategies.

This study systematically analyzed KOA-related differentially expressed genes (DEGs) based on publicly available sample data using bioinformatics approaches. Key hub genes were identified through Protein-protein interaction (PPI) network construction, Gene ontology (GO) semantic similarity analysis, and Gene Set Variation Analysis (GSVA) pathway enrichment. Additionally, immune infiltration analysis was conducted to explore the association between these genes and the immune microenvironment. Finally, *in vitro* cell models combined with real-time quantitative PCR (RT-qPCR) experiments were used to validate the expression of hub genes. This study aims to elucidate the potential mechanisms of these genes in the pathogenesis of KOA and to provide a theoretical basis for identifying potential biomarkers and developing targeted therapeutic strategies.

## Methods

### Data collection

The transcriptomic data used in this study were obtained from the Gene Expression Omnibus (GEO) database (https://www.ncbi.nlm.nih.gov/geo/), specifically from the high-throughput sequencing dataset GSE114007. The dataset includes annotated files based on the GPL11154 and GPL18573 platforms and comprises samples from 20 patients with KOA and 18 healthy controls.

### Differentially expressed genes screening

Differential expression analysis of the GSE114007 dataset was performed using the limma package in R (v4.4.0), with screening thresholds set at adjusted *p*-value (adj. p < 0.05) and |log_2_FoldChange| > 1. Volcano plots and heatmaps were generated using the ggplot2 and pheatmap packages, respectively, to visualize gene expression differences.

### Gene ontology (GO) and Kyoto Encyclopedia of Genes and Genomes (KEGG) pathway enrichment analysis

DEGs were subjected to GO enrichment analysis and KEGG pathway enrichment analysis using the clusterProfiler package (version 3.14.3) in RStudio. Multiple-testing correction was applied using the Benjamini–Hochberg false discovery rate (FDR) method, and pathways with adjusted *p* < 0.05 were considered significantly enriched. GO analysis covered three categories: Biological Process (BP), Cellular Component (CC), and Molecular Function (MF). The enrichment results were visualized using the ggplot2 package in R.

### Screening of hub genes through Gene Ontology-based semantic similarity

GO-based semantic similarity analysis was performed on DEGs using the GOSemSim package (version 2.30.0), with a threshold score greater than 0.4 to identify genes with high functional similarity for subsequent analysis (Xue et al., 2023).

### Construction of protein-protein interaction network and identification of hub genes

A PPI network of the DEGs was constructed using the STRING database (https://string-db.org/) with a confidence score threshold of 0.7 (high confidence), and disconnected nodes were removed. The resulting network was imported into Cytoscape software, key modules were identified using MCODE (the Molecular Complex Detection) following filter criteria: degree cut-off = 2; node score cut-off = 0.2; k-core = 2; and max depth = 100. Hub genes were further identified using the CytoHubba plugin (version 0.1).

### Gene set variation analysis

GSVA was performed using the GSVA package (version 4.12.0) in Bioconductor to evaluate the activity changes of hub gene-related pathways across differentially expressed samples. KEGG pathway gene sets were used for the analysis, and pathways with both *p*-values and *q*-values <0.05 were considered significantly enriched, revealing the potential functional roles of hub genes in the pathogenesis of KOA.

### Immune infiltration analysis

Immune cell infiltration was assessed using the CIBERSORT algorithm to estimate the relative proportions of 22 immune cell types within each sample. The analysis was conducted based on expression data, comparing KOA samples with healthy controls. Correlations between hub genes and immune cell infiltration levels were calculated using Spearman’s correlation coefficient. Immune cell types showing significant differences between the two groups were identified using the thresholds of |R| > 0.5 and *p* < 0.05.

### Cell culture and treatment

*In vitro* experiments were conducted using the human chondrocyte cell line CHON-001 (CRL-2846, ATCC, Manassas, VA, United States). Cells were cultured in RPMI-1640 medium supplemented with 10% fetal bovine serum and 1% penicillin/streptomycin at 37 °C in a humidified atmosphere containing 5% CO_2_. The cells were divided into two groups: a control group maintained under standard conditions, and a treatment group stimulated with 10 ng/mL IL-1β for 24 h to establish an inflammation-induced cartilage injury model (Yang et al., 2019a).

### RT-qPCR

Total RNA was extracted using an RNA extraction kit (K157002, Thermo Fisher Scientific, United States), and reverse-transcribed into complementary DNA (cDNA). RT-qPCR was carried out on a ViiA™ 7 Real-Time PCR System under the following cycling conditions: 95 °C for 30 s, followed by 40 cycles of 95 °C for 10 s and 60 °C for 32 s β-actin was used as an internal control, and relative gene expression was calculated using the 2^−ΔΔCt^ method. Each sample was analyzed in triplicate, and the average value was used for further analysis.

### Statistical analysis

Statistical analyses were conducted using SPSS software (version 23.0; IBM, Armonk, NY, United States). All data were presented as mean ± standard deviation (SD). The Shapiro–Wilk test confirmed normal distribution of the data. Differences between two groups were assessed using the independent samples *t*-test. For analyses involving multiple comparisons, *p*-values were adjusted using the Benjamini–Hochberg false discovery rate (FDR) method, and adjusted *p* < 0.05 was considered statistically significant.

## Results

### Identification of differentially expressed genes

**FIGURE 1:**
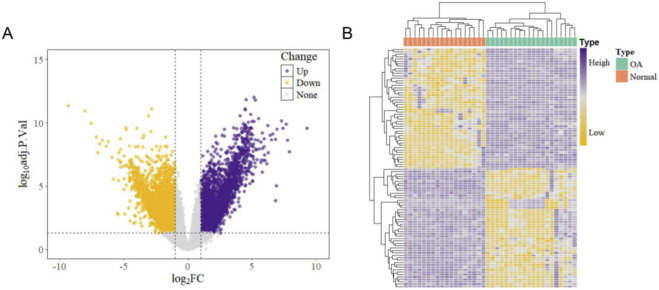
Screening for DEGs **(A)** Volcano plot showing the distribution of DEGs; yellow indicates downregulated genes, and purple represents upregulated genes. **(B)** Heatmap illustrating the expression patterns of DEGs; purple indicates high expression levels, while yellow indicates low expression levels. DEGs: differentially expressed genes.

Through the analysis of the GSE114007 dataset from the GEO database, a total of 3,290 upregulated genes and 2,536 downregulated genes were identified (Supplementary Tables S1,S2). The distribution of DEGs was visualized using a volcano plot and a heatmap, providing an intuitive representation of the gene expression differences between patients with KOA and healthy controls (Figure 1).

### Gene Ontology and Kyoto Encyclopedia of Genes and Genomes pathway enrichment analysis

**FIGURE 2:**
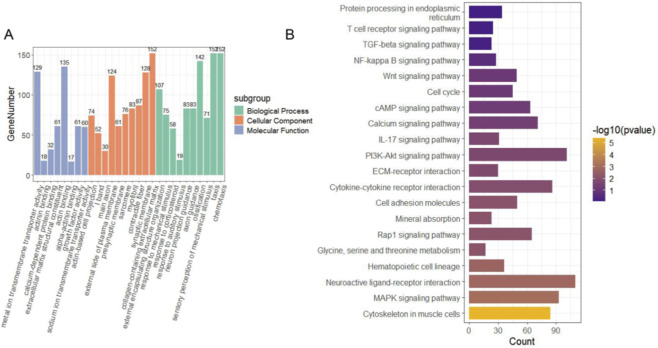
GO and KEGG pathway enrichment analysis **(A)** GO enrichment analysis showing the categories of biological process (BP), cellular component (CC), and molecular function (MF). **(B)** KEGG pathway analysis illustrating significantly enriched signaling pathways associated with the DEGs.

GO functional annotation and KEGG pathway enrichment analysis were performed on the identified DEGs. Detailed GO and KEGG enrichment results for all differentially expressed genes are available in Supplementary Tables S3,S4. The results of the GO analysis (Figure 2A) showed that these genes were primarily enriched in BP such as protein complex assembly, ECM organization, receptor complex formation, and transmembrane transport. In terms of MF, the DEGs were significantly enriched in activities related to metal ion and sodium ion transmembrane transport. Furthermore, KEGG pathway analysis (Figure 2B) indicated that the DEGs were significantly enriched in several signaling pathways associated with inflammation, cell proliferation, and immune regulation, including the mitogen-activated protein kinase (MAPK) signaling pathway, ECM-receptor interaction, cytokine-receptor interaction, and phosphoinositide 3-kinase-Akt (PI3K-Akt) signaling pathway.

### Identification of hub genes

**FIGURE 3:**
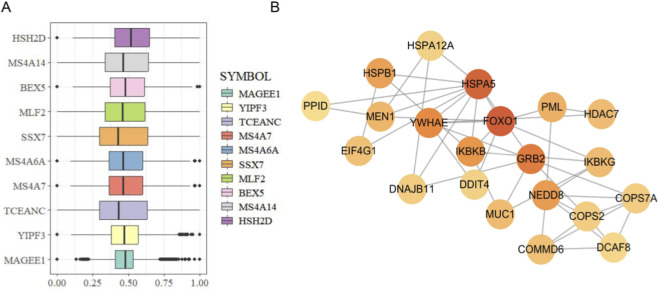
Identification of hub genes **(A)** Boxplot showing the top 10 genes with the highest GO-based semantic similarity scores. **(B)** PPI network analysis highlighting hub genes with high connectivity, which may play key roles in the pathogenesis of KOA. The node color represents the degree of connectivity, with yellow indicating lower connectivity and red indicating higher connectivity.

A total of 340 functionally related genes were identified through GO semantic similarity analysis (Supplementary Table S5), and the top 10 genes with the highest semantic similarity scores were visualized (Figure 3A). Subsequently, a PPI network of the DEGs was constructed using the STRING database. The MCODE plugin in Cytoscape was applied to identify densely connected modules, and the CytoHubba plugin was used to screen hub genes with high connectivity within the network. As shown in Figure 3B, genes such as HSPA5, FOXO1, and YWHAE exhibited high degrees of connectivity and were located at the core of multiple high-scoring modules, suggesting their potential central roles in the molecular regulatory network of KOA.

### Gene set variation analysis

**FIGURE 4:**
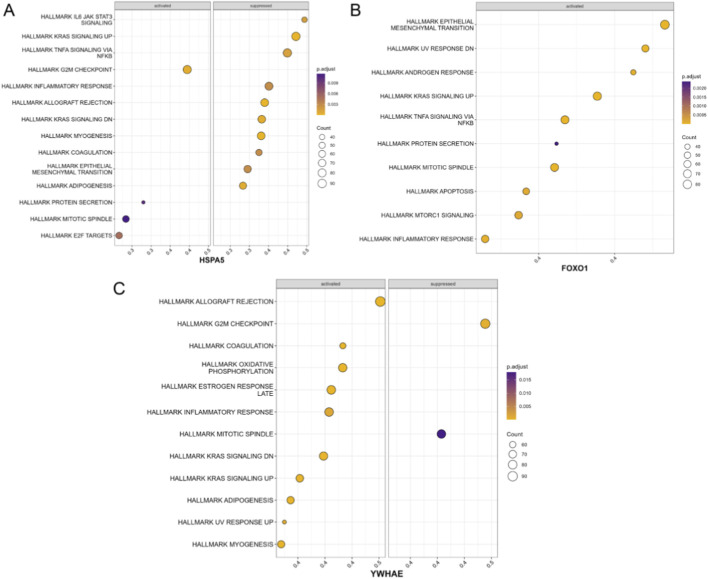
Enrichment of signaling pathways associated with hub genes based on GSVA. **(A–C)** Dot plots illustrating GSVA results for HSPA5 **(A)**, FOXO1 **(B)**, and YWHAE **(C)**, showing significantly enriched signaling pathways across different sample groups.

Functional analysis of the hub genes revealed that HSPA5, FOXO1, and YWHAE were significantly enriched in multiple signaling pathways between KOA patients and healthy controls (Figures 4A–C). GSVA was performed using the Bioconductor GSVA package to calculate pathway enrichment scores for each hub gene across different samples (Supplementary Tables S6-S8). The results showed that these genes were significantly enriched in several key pathways, including the nuclear factor kappa B (NF-κB) signaling pathway, the Janus kinase-signal transducer and activator of transcription (JAK-STAT) pathway, and the MAPK signaling pathway.

### Immune infiltration analysis

**FIGURE 5:**
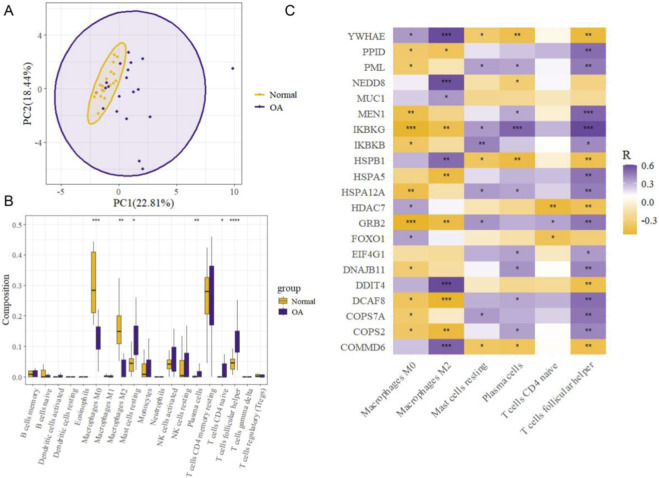
Immune infiltration analysis **(A)** PCA plot illustrating overall differences in immune cell infiltration profiles between KOA patients and healthy controls. **(B)** Boxplot showing the relative proportions of 22 immune cell types in KOA and control groups. Significant differences were observed in six immune cell types. **(C)** Heatmap displaying the correlations between hub genes and immune cell types. Color indicates the Spearman correlation coefficient (*R*), and asterisks denote significance levels (*p* < 0.05, *p* < 0.01, *p* < 0.001).

Principal component analysis (PCA) revealed a partial separation trend between the KOA and normal groups along the PC1 and PC2 dimensions (Figure 5A). This suggests modest but not definitive differences in overall immune cell infiltration profiles, which may be influenced by the limited sample size. To obtain a more detailed view, CIBERSORT analysis was performed and revealed significant differences in immune cell composition between KOA patients and healthy controls. Six immune cell types showed statistically significant variation, including M0 macrophages, M2 macrophages, resting mast cells, plasma cells, naïve CD4^+^ T cells, and follicular helper T cells (Figure 5B). Further analysis displayed correlations between the infiltration levels of these immune cell types and the expression of hub genes. For example, YWHAE, NEDD8, and HSPB1 were positively associated with M2 macrophage infiltration, but negatively correlated with resting mast cells and follicular helper T cells (Figure 5C). The underlying data corresponding to Figure 5, including the immune cell infiltration matrix and correlation analysis results, are provided in Supplementary Table S9.

### Validation of hub gene expression in IL-1β-stimulated CHON-001 cells

**FIGURE 6:**
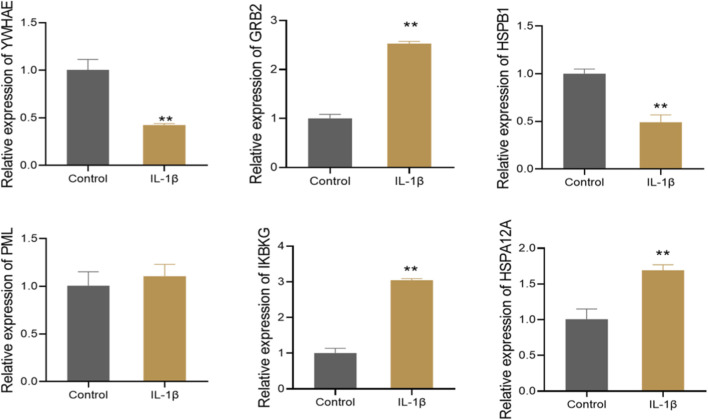
Validation of hub gene expression in IL-1β-stimulated CHON-001 cells. **P < 0.01 vs. Control group.

To further confirm the bioinformatics predictions, we selected six genes (GRB2, IKBKG, HSPA12A, YWHAE, HSPB1, and PML) for experimental validation. These genes were prioritized as hub genes with central roles in the PPI network and semantic similarity analysis. To simulate the pathological state of chondrocytes in KOA, CHON-001 cells were treated with IL-1β. As shown in Figure 6, the expression levels of YWHAE and HSPB1 were significantly downregulated, while GRB2, IKBKG, and HSPA12A were markedly upregulated following IL-1β stimulation. No significant changes were observed in the expression of PML. These findings are consistent with the predictions from the bioinformatics analysis. The complete RT-qPCR validation data supporting Figure 6 are presented in Supplementary Table S10.

## Discussion

This study revealed the potential mechanisms of hub genes in KOA through bioinformatics analysis. Differential expression analysis identified 3,290 upregulated and 2,536 downregulated genes in the cartilage of KOA patients, with HSPA5, FOXO1, YWHAE, and other genes identifiedas as hub genes. Functional enrichment analyses indicated that these genes are involved in ECM organization, protein complex assembly, and transmembrane signaling, as well as MAPK, PI3K-Akt, and ECM-receptor pathways—processes known to be central to cartilage degradation and joint inflammation.

KOA is a common musculoskeletal disorder that primarily affects the elderly, characterized by marginal bone spurs, cartilage erosion, and biochemical and morphological changes in the synovium and joint capsule (Brosseau et al., 2017). Treatment options for KOA include medications, surgery, and physical therapy, with current clinical guidelines recommending non-pharmacological and non-surgical conservative strategies as the primary approach (Kan et al., 2019). Although existing treatments focus on symptom relief and slowing disease progression, the complexity of its pathological processes means that treatment efficacy may gradually diminish as the disease progresses (Giorgino et al., 2023). In addition, the assessment of KOA is still based on radiological examinations and subjective pain evaluations. Therefore, identifying potential biomarkers and underlying mechanisms of KOA is crucial. This will not only deepen our understanding of the disease pathogenesis but also provide a theoretical foundation for early diagnosis, prognostic evaluation, and the development of targeted therapeutic strategies.

Previous studies have confirmed a positive correlation between the progressive degeneration of articular cartilage and synovial inflammation (Ene et al., 2015; Moravek et al., 2023). Pro-inflammatory cytokines such as IL-1β, TNFα, and IL-6 accelerate cartilage degradation by inducing the release of MMPs, disrupting the ECM, and promoting the production of additional inflammatory mediators (Meehan et al., 2021). Chemokines like CCL2, CCL3, CCL4, CCL5, and CXCL12 further exacerbate the inflammatory response and tissue damage in KOA by recruiting inflammatory cells or modulating immune responses (Bhavsar et al., 2015; Pierzchala et al., 2011). Additionally, the NF-κB signaling pathway plays a critical role in this process (Yang et al., 2019b), suggesting that these factors hold potential as therapeutic targets for OA treatment. In our study, several hub genes with significant network topological features were identified through GO-based semantic similarity and PPI network analysis, including HSPA5, FOXO1, and YWHAE. Previous studies have shown that reduced HSPA5 signaling is associated with alleviated cartilage damage in a rabbit KOA model. HSPA5 contributes to maintaining iron homeostasis in cartilage, thereby preventing iron dysregulation in chondrocytes and mitigating ECM degradation (Meng et al., 2024). In addition, FOXO1 signaling has been reported to alleviate OA progression by promoting autophagy in chondrocytes (Wu et al., 2024). These findings suggest these hub genes may play important roles in the pathogenesis of KOA.

Immune infiltration analysis revealed altered proportions of several immune cell types in KOA samples, including M0 and M2 macrophages, plasma cells, and mast cells. Correlation analysis showed associations between hub gene expression and immune cell infiltration, particularly the positive correlation between YWHAE, HSPB1, and M2 macrophages—suggesting potential immunomodulatory roles (Rosini et al., 2023; Pan et al., 2022). However, these associations are correlative, and the precise mechanisms by which hub genes influence immune cell behavior remain undefined. Future studies should explore these interactions using gene perturbation combined with immune co-culture systems and cytokine profiling to establish causal relationships.

Finally, we validated the mRNA expression levels of selected hub genes using the IL-1β-stimulated CHON-001 cell model. Compared with the control group, YWHAE and HSPB1 were significantly downregulated, while GRB2, IKBKG, and HSPA12A were significantly upregulated, further supporting their potential involvement in the pathogenesis and progression of KOA. Nonetheless, this model does not fully replicate the complex mechanical and degenerative microenvironment of KOA, and our validation was limited to mRNA levels without assessing protein expression or functional phenotypes. Additional studies involving protein-level validation, signaling pathway interrogation, and phenotypic assays are necessary to clarify the roles of these genes in KOA.

From a translational perspective, several hub genes identified in this study may have clinical relevance. HSPB1 and HSPA5, in particular, are involved in druggable pathways such as stress response and NF-κB signaling (Liang et al., 2023; Li et al., 2023). Although we did not assess druggability or therapeutic potential directly, follow-up studies will explore these genes using pharmacological databases (e.g., DrugBank, ChEMBL) and small-molecule screening. Moreover, while these genes show promise as potential biomarkers, we have not evaluated their diagnostic performance. Future validation in independent cohorts with receiver operating characteristic (ROC) curve analysis will be essential to determine sensitivity, specificity, and clinical utility.

This study has several limitations that should be acknowledged. First, the bioinformatics analyses relied on a single GEO dataset with a relatively small sample size and lacked detailed clinical metadata, preventing stratified analyses by disease severity or patient heterogeneity. Second, the experimental validation was restricted to an IL-1β–induced chondrocyte model, which can only partially mimic the inflammatory environment of KOA and does not fully capture the mechanical and chronic degenerative features of the disease. Moreover, validation was performed only at the mRNA level without confirmation at the protein level or functional assays such as gene knockdown/overexpression, which are needed to clarify causal roles. Third, our interpretations were largely based on correlations, with limited mechanistic exploration of hub gene–immune cell interactions. Finally, the absence of external validation in independent cohorts or *in vivo* models restricts generalizability. Future work integrating larger, clinically annotated datasets, protein-level and functional experiments, and animal studies will be necessary to confirm these findings and assess their translational potential.

In summary, this study integrated bioinformatics analyses with *in vitro* validation to identify key hub genes and their potential roles in the immune microenvironment of KOA. Despite the acknowledged limitations, our findings provide new insights into the molecular mechanisms underlying KOA and suggest promising directions for biomarker discovery and targeted therapy. Future research involving larger independent cohorts, protein-level and functional validation, and *in vivo* models will be necessary to strengthen these observations and accelerate their translation into clinical practice.

## Data Availability

The raw data supporting the conclusions of this article will be made available by the authors, without undue reservation.

## Abbreviations

KOA, Knee osteoarthritis; OA, osteoarthritis; DEGs, differentially expressed genes; PPI, protein-protein interaction; GSVA, gene set variation analysis; CIBERSORT, cell-type identification by estimating relative subsets of RNA transcripts; GEO, Gene Expression Omnibus; GO, Gene Ontology; KEGG, Kyoto Encyclopedia of Genes and Genomes; IL-1β, interleukin-1 beta; qRT-PCR, quantitative real-time polymerase chain reaction; SD, standard deviation; ECM, extracellular matrix; MAPK, mitogen-activated protein kinase; HSPA5, heat shock protein family A member 5; FOXO1, forkhead box O1; GRB2, growth factor receptor-bound protein-2; PML, promyelocytic leukemia protein; IKBKG, inhibitor of nuclear factor kappa B kinase regulatory subunit gamma; COPS2, COP9 signalosome subunit 2; DCAF8, DDB1- and CUL4-associated factor 8.
